# Pharmacotranscriptomic Analysis Reveals Novel Drugs and Gene Networks Regulating Ferroptosis in Cancer

**DOI:** 10.3390/cancers12113273

**Published:** 2020-11-05

**Authors:** Haitang Yang, Liang Zhao, Yanyun Gao, Feng Yao, Thomas M. Marti, Ralph A. Schmid, Ren-Wang Peng

**Affiliations:** 1 Division of General Thoracic Surgery, Department of BioMedical Research (DBMR), Inselspital, Bern University Hospital, University of Bern, 3008 Bern, Switzerland; haitang.yang@dbmr.unibe.ch (H.Y.); liang.zhao@dbmr.unibe.ch (L.Z.); yanyun.gao@dbmr.unibe.ch (Y.G.); thomas.marti@insel.ch (T.M.M.); 2 Department of Thoracic Surgery, Shanghai Chest Hospital, Shanghai Jiao Tong University, Shanghai 200030, China; feng.yao@shchest.org

**Keywords:** ferroptosis, pharmacotranscriptomics, gene signature, ferroptosis-based therapy, HDAC, IDH mutation, small-cell lung cancer, precision oncology

## Abstract

**Simple Summary:**

Ferroptosis is increasingly recognized as a promising avenue for cancer therapy, while the biomarkers that predict the sensitivity and/or resistance of ferroptosis and the molecular mechanisms that can be therapeutically exploited to modulate ferroptosis are not yet known. Here, we perform an integrated pharmacotranscriptomic analysis to systematically identify compounds and gene networks that regulate ferroptosis. Our results provide mechanistic insights into the deregulation of ferroptosis in cancer and suggest new approaches for ferroptosis-based cancer therapy.

**Abstract:**

(1) Background: Ferroptosis is an apoptosis-independent cell death program implicated in many diseases including cancer. Emerging evidence suggests ferroptosis as a promising avenue for cancer therapy, but the paucity of mechanistic understanding of ferroptosis regulation and lack of biomarkers for sensitivity to ferroptosis inducers have significantly hampered the utility of ferroptosis-based therapy. (2) Methods: We performed integrated dataset analysis by correlating the sensitivity of small-molecule compounds (*n* = 481) against the transcriptomes of solid cancer cell lines (*n* = 659) to identify drug candidates with the potential to induce ferroptosis. Generalizable gene signatures of ferroptosis sensitivity and resistance are defined by interrogating drug effects of ferroptosis inducers (*n* = 7) with transcriptomic data of pan-solid cancer cells. (3) Results: We report, for the first time, the comprehensive identification of drug compounds that induce ferroptosis and the delineation of generalizable gene signatures of pro- and anti-ferroptosis in pan-cancer. We further reveal that small cell lung cancer (SCLC) and isocitrate dehydrogenase (*IDH1/2*)-mutant brain tumors show enrichment of pro-ferroptosis gene signature, suggesting a unique vulnerability of SCLC and *IDH*-mutant tumors to ferroptosis inducers. Finally, we demonstrate that targeting class I histone deacetylase (HDAC) significantly enhances ferroptotic cell death caused by Erastin, an ferroptosis inducer, in lung cancer cells, revealing a previously underappreciated role for HDAC in ferroptosis regulation. (4) Conclusions: Our work reveals novel drug compounds and gene networks that regulate ferroptosis in cancer, which sheds light on the mechanisms of ferroptosis and may facilitate biomarker-guided stratification for ferroptosis-based therapy.

## 1. Introduction

Escape from cell death is fundamental for cancer development. Ferroptosis is a new form of programmed cell death genetically and biochemically different from apoptosis, necroptosis and autophagy-mediated death programs [1,2]. Physiologically activated by metabolic accumulation of lipid peroxides, ferroptosis is frequently dysregulated in cancers and confers a key mechanism of therapy resistance, highlighting ferroptosis as a promising avenue for cancer therapy [3,4].

Ferroptosis is negatively regulated by a lipid radical-specific antioxidant defense system such as glutathione peroxidase 4 (GPX4) that hydrolyzes lipid hydroperoxides and thereby protects cells from ferroptosis [5]. Antagonizing GPX4 with small molecules, such as the rat sarcoma viral oncogene homolog (RAS)-selective lethal 3 (RSL3), efficiently induces ferroptosis [1]. The reductase activity of GPX4 requires the co-factor glutathione (GSH), an abundant cellular tripeptide consisting of glycine, glutamate, and cysteine, as an electron donor to reduce lipid hydroperoxides. GSH synthesis depends on intracellular availability of the precursor cysteine that is mainly generated from the reduction of cystine; thus, cystine depletion also induces ferroptosis [2]. As cysteine is imported extracellularly via the sodium-independent cystine/glutamate antiporter system xc-, a heterodimer consisting of a heavy chain (4F2, also known as SLC3A2) and a light chain (xCT or SLC7A11) [2], targeting xCT/SLC7A11, e.g., by the small-molecule inhibitor Erastin, restrains cystine supply and provokes ferroptosis [1,2]. 

In addition to GPX4 and xCT/SLC7A11, the ferroptosis suppressor protein 1 (FSP1), also known as apoptosis-inducing factor mitochondria associated 2 (AIFM2), plays a GPX4-independent anti-ferroptosis role [6,7] and combined inhibition of GPX4 and FSP1 synergistically enhances ferroptosis [6,7]. Moreover, recent studies have identified other factors involved in ferroptosis regulation [8,9,10,11,12]. The KEAP1 (Kelch ECH associating protein 1) and NRF2 (nuclear factor erythroid 2-related factor 2) are well-characterized in regulating cytoprotective response to oxidative stress [13]. KEAP1 is a repressor of the transcription factor NRF2 by directly binding to and inducing NRF2 degradation via the ubiquitin-proteasome pathway. Genetic alterations that inactivate *KEAP1*, common in lung cancer [14], lead to NRF2 activation and subsequently expression of NRF2 target genes to promote antioxidant defense, indicating that the KEAP1-NRF2 axis is closely related to ferroptosis [15,16]. Despite the progress, our understanding of the gene network underlying ferroptosis dysregulation in cancer remains poor.

Ferroptosis dysregulation contributes to cancer pathogenesis [10,17], unveiling ferroptosis as a promising anticancer strategy [8] or to overcome therapeutic resistance [18]. However, ferroptosis inducers alone have only achieved limited success, highlighting the need for further stratification and for identification of additional targets to improve the efficacy of ferroptosis-based therapy. Recent studies have shown that the Hippo pathway and epithelial-to-mesenchymal transition (EMT) are associated with sensitivity to ferroptosis [8,18,19]. In this study, we performed integrative dataset analysis by correlating sensitivity profiling of a large drug library (*n* = 481) across transcriptomic gene expression of a huge cohort of solid cancer cell lines, and revealed, for the first time, novel drug candidates with the potential to induces ferroptosis in cancer and gene networks associated with cancer cell response to ferroptosis inducers. Systematic identification of ferroptosis-inducing agents and of generalizable gene signatures of ferroptosis shed mechanistic light on ferroptosis regulation and may facilitate the utility of ferroptosis as the avenue for cancer therapy.

## 2. Results

### 2.1. Systematic Correlation Identifies Cancer Drugs with the Potential to Induce Ferroptosis

**Figure 1:**
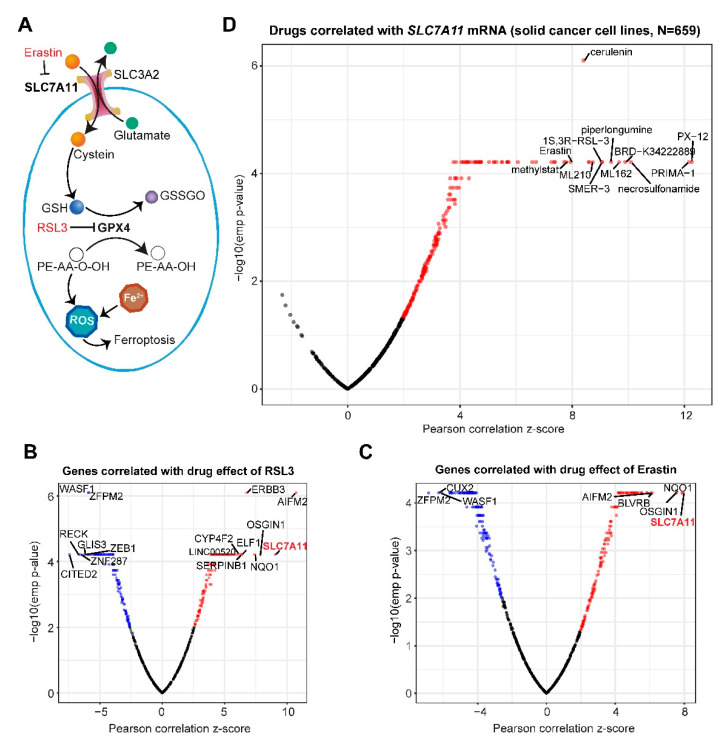
Comprehensive pharmacogenomic analysis identifies cancer drugs with the potential to induce ferroptosis. (**A**), Schematic of ferroptosis regulation. GSSGO, oxidized glutathione disulfide; PE-AA-O-OH and PE-AA-OH, phosphatidylethanolamine (PE), arachidonoyl (AA), hydroperoxide group (-O-OH). GPX4 protects against ferroptosis by catalyzing GSH and toxic PE-AA-OOH into oxidized GSH (GSSGO) and nontoxic PE-AA-OH. (**B**,**C**), Gene clusters associated with sensitivity to RSL3 (**B**) and Erastin (**C**) across solid cancer cell lines (*n* = 659). Blue dots indicate the significantly negatively correlated genes while the red the positively correlated ones. The most significantly correlated genes were highlighted. Here, a negative correlation indicates the association of a larger AUC (area under the curve) area with lower gene expression and vice versa. (**D**), Comprehensive analysis of cancer drugs (*n* = 481) whose activities are significantly correlated with *SLC7A11* gene expression across the solid cancer cell lines (*n* = 659). Red dots indicate drugs whose effectiveness (AUC value) positively correlates with *SLC7A11* mRNA.

Ferroptosis is negatively regulated by the SLC7A11-GPX4 signaling axis (Figure 1A). To systematically identify cancer drugs that modulate ferroptosis response of cancer cells, we correlated the sensitivity profiling of a previously curated small-molecule compound library (*n* = 481) containing FDA-approved drugs, clinical candidates and those interrogating important targets and/or cellular processes in cancer, against the transcriptomes of a cohort of pan-cancer cell lines (*n* = 659) [20]. This analysis revealed that gene expression (mRNA) of *SLC7A11* most strongly positively correlates with the area under the curve (AUC), a measure of drug sensitivity determined by fitted concentration-response curves, of both RSL3 (Pearson correlation z-score = 9.05; *p* = 6.10 × 10^−05^) and Erastin (Pearson correlation z-score = 7.94; *p* = 6.10 × 10^−05^), two classical ferroptosis-inducing agents (Figure 1B,C). This observation indicates that cancer cells with higher *SLC7A11* mRNA levels have greater AUC values, and thus are less sensitive or more resistant to ferroptosis induction, which is consistent with previous studies reporting that SLC7A11 is a core negative regulator of ferroptosis and increasingly appreciated as a therapeutic target in cancers [1,2]. Interestingly, both RSL3 and Erastin showed no significant correlation with *GPX4* or *SLC3A2* (data not shown), which might be due to the high abundance of the two genes in cancer cells. 

Next, we systematically correlated sensitivity data (determined by AUC) of the small-molecule compounds (*n* = 481) with *SLC7A11* gene expression across the entire cancer cell line cohort (*n* = 659). This analysis identified a total of 139 drug candidates whose AUC values significantly (empirical *p*-value < 0.01) positively correlate with *SLC7A11* (Figure 1D; Table S1), including ML162, ML210, RSL3, PX-12, PRIMA-1, Piperlongumine, and Erastin that were previously shown to trigger ferroptosis [21], validating the robustness of the systematic correlation study and the accountability of our results. 

**Figure 2:**
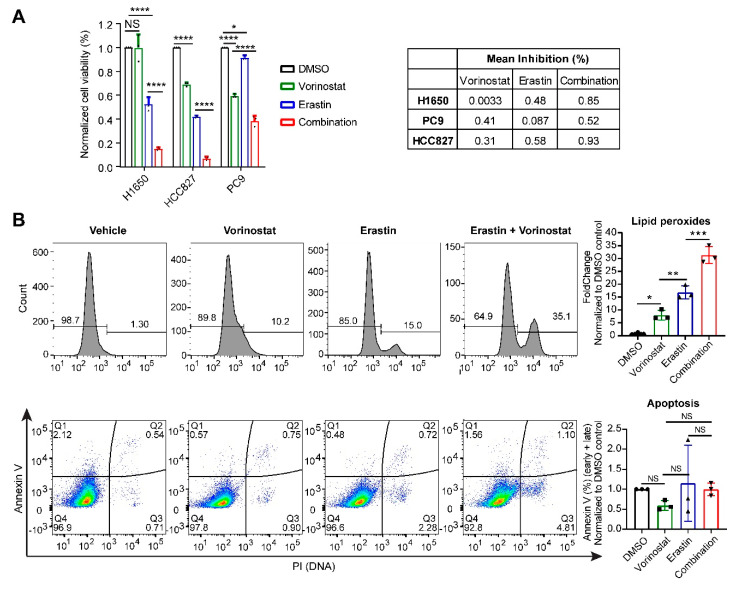
HDAC (histone deacetylase) inhibition enhances antitumor efficacy of a ferroptosis inducer in lung cancer cells. (**A**), Non-small cell lung cancer cells (H1650, HCC827, PC9) were treated with Vorinostat (HDACi; 1 µM), Erastin (1 µM), alone and in combination (Vorinostat [1 µM] plus Erastin [1 µM]) for 72 h. Cell viability was quantified by counting cell number. (**B**), Flow cytometry-based analysis of lipid peroxides (upper panel) and apoptosis (middle panel) in H1650 cells treated for 24 h with Vorinostat [1 µM], Erastin [1 µM] and alone and in combination. Representative figures (upper and middle panels) and their quantification (lower panel) were shown (*n* = 3 biological repeats). PI: Propidium iodide. * *p*  <  0.05, ** *p*  <  0.01, *** *p*  <  0.001, **** *p*  <  0.0001 by one-way ANOVA (*n* = 3). NS: not significant.

The pattern by which these drugs cluster in the systematic correlation analysis suggests that they may share the mode of action in regulating ferroptosis [20]. Importantly, our analysis revealed a set of drug candidates with ferroptosis-activating potential were neither previously reported nor recorded by the FerrDb (http://www.zhounan.org/ferrdb/), a manually curated dataset elaborating on ferroptosis (Table S1). Analyzing the annotated targets of the identified compounds revealed that several pathways, including ROS (reactive oxygen species) modulation, fatty acid biosynthesis regulation, MDM2-p53 signaling, receptor tyrosine kinases, NAMPT (nicotinamide phosphoribosyltransferase), ubiquitin-proteasome and, particularly, PI3K-AKT1-mTOR and epigenetic regulators, are enriched (Table S2), suggesting that these signaling cascades may be involved in ferroptosis deregulation in cancer. Supporting our findings, recent studies have demonstrated that p53 and mTOR play essential roles in regulating ferroptosis [17,22]. Importantly, our data implicated an unexpected role for class I histone deacetylase (HDAC) family in ferroptosis regulation (Table S2). To verify our finding, we treated NSCLC cells (H1650, PC9 and HCC827) with Erastin and Vorinostat, a clinically-approved class I HDAC inhibitor, alone or in combination, which showed that the presence of Vorinostat significantly enhances the anti-proliferative effect of Erastin (Figure 2A). Importantly, combined treatment with Vorinostat and Erastin significantly increased the lipid peroxides level in lung cancer cells compared to single agents. In sharp contrast, Vorinostat in combination with Erastin had similar effects on apoptosis as single drugs (Figure 2B), suggesting that HDAC inhibition combined with Erastin indeed induces ferroptosis. Notably and consistently with our findings, previous studies showed that class I HDAC inhibitors induce ROS-dependent cell death although the underlying mechanisms were not clear [23,24,25]. 

### 2.2. Gene Networks Associated with Ferroptosis Sensitivity and Resistance in Pan-Cancer

Cancer cells show high heterogeneity in response to ferroptosis-based therapeutics [8], highlighting the need for further stratification. We therefore sought to delineate the gene networks linked with ferroptosis sensitivity and resistance in cancer cells. To generalize the results and minimize drug-specific and potential off-target effects, multiple established ferroptosis-inducing molecules, namely ML162, ML210, Necrosulfonamide, PRIMA, PX-12, RSL3, and Erastin whose sensitivity most significantly correlate with *SLC7A11* expression (Figure 1D) were integrated in our analysis. Correlating drug sensitivity data with basal gene expression of pan-solid cancer cell lines (*n* = 659) revealed that, as expected (Figure 1A–C), *SLC7A11* re-appeared as of one of the top hits with their expression most significantly positively correlated with the AUC values of all seven drugs (Figure S1A–E), reinforcing the robustness of our approach.

**Figure 3:**
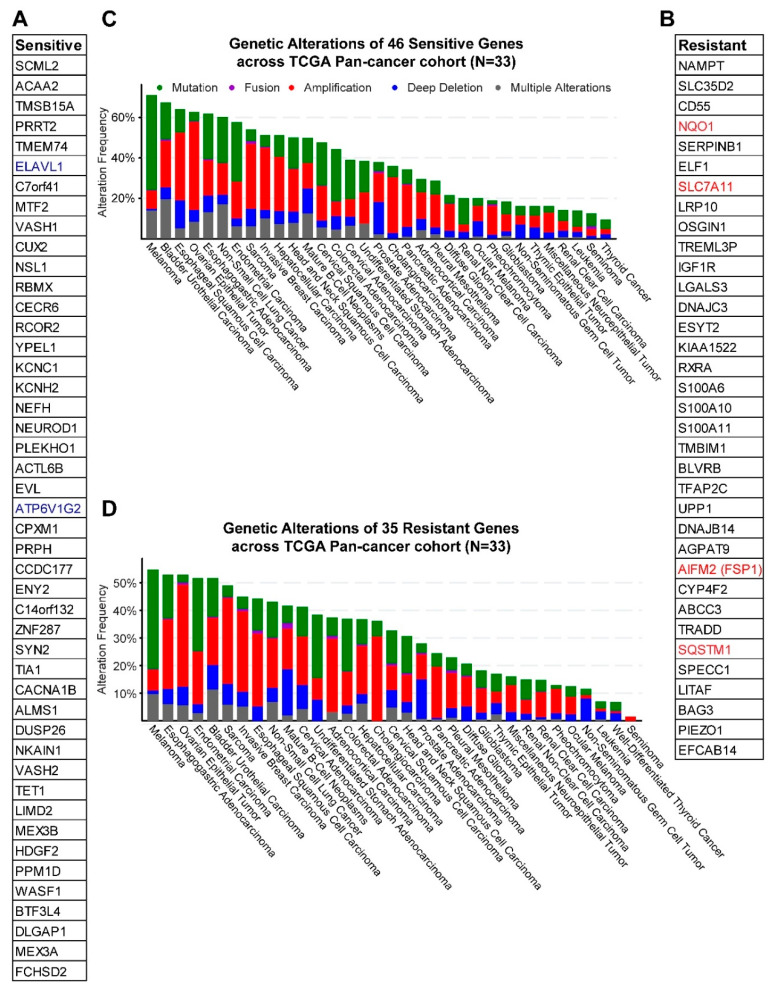
Gene networks linked with ferroptosis sensitivity and resistance in pan-cancer. (**A**,**B**), Gene clusters in ferroptosis sensitive (**A**) and resistant (**B**) groups. Blue and red colors indicate genes previously reported or curated by FerrDb. (**C**,**D**), Genetic alterations of the genes in ferroptosis sensitive (**C**) and resistant (**D**) groups across a pan-cancer cohort in TCGA.

The genes significantly (empirical *p*-value < 0.01) correlated with the selected drugs fell into the sensitive group (high expression linked with increased ferroptosis susceptibility or decreased AUC), containing those negatively correlated with drug effects (AUC), and the resistant group whereby the expression of genes positively correlated with AUC values. Notably, ZEB1, previously shown to be a marker for sensitivity to ferroptosis [18], is negatively correlated with several ferroptosis inducers (Figure 1B and Figure S1A,B), while FSP1/AIFM2, an anti-ferroptotic regulator independent of GPX4 [6,7], is in the resistant group (Figure 3 and Figure S1A,B). The coverage of previously identified ferroptosis regulators reiterates the validity of our results.

To curate a generalized gene signature for ferroptosis sensitivity, we focused on the candidates common to all tested drugs. By setting a stringent threshold at an empirical *p*-value < 0.01, we finally delineated a set of 46 and 35 genes linked with ferroptosis sensitivity and resistance, respectively (Figure 3A,B). Supporting our results, two genes (*ELAVL1* and *ATP6V1G2*) in the sensitive group (Figure 3A) and four (*SLC7A11*, *FSP1*/*AIFM2*, *NQO1* and *SQSTM1*) in the resistant group (Figure 3B) were previously reported or curated by the FerrDb (http://www.zhounan.org/ferrdb/) [26] that fulfill the same function as assigned by our study. Notably, the functional link between ferroptosis and the vast majority of these genes (44 of 46 in the sensitive and 31 of 35 in the resistant group) have not been shown previously. The interaction network and pathways engaged by the identified genes were shown in Figure S2A,B. Interestingly, genes in both groups are frequently altered, despite varied degrees in different cancers (Figure 3C,D), which may enable further stratifications for ferroptosis-based therapy. Importantly, some genes (*NAMPT*, *IGF1R*, *CYP4F2*, *BLVRB*) in the resistant group are therapeutically exploitable according to the druggable genome database (http://dgidb.org/). 

**Figure 4:**
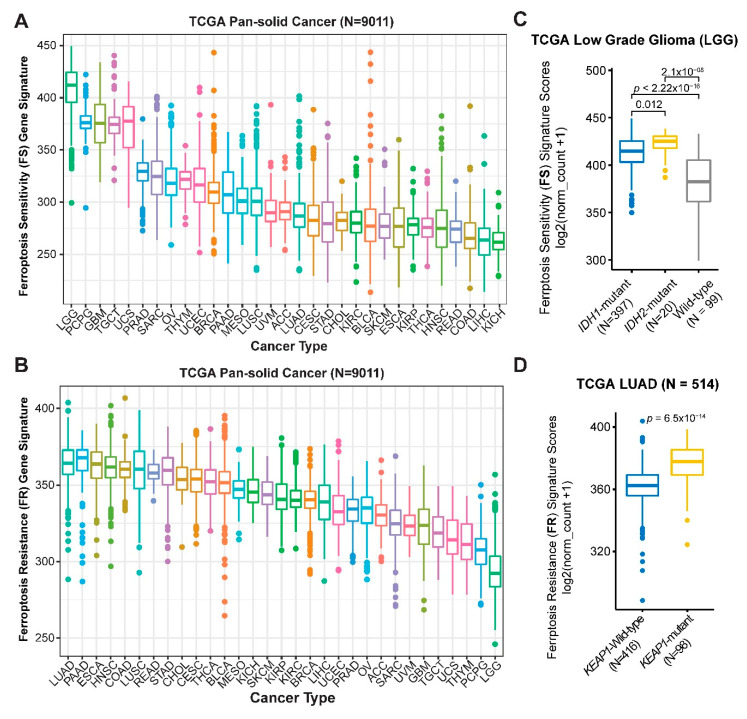
Generalized gene signatures were predictive of ferroptosis sensitivity and resistance. (**A**,**B**), Prospective analysis of ferroptosis sensitivity (FS) (**A**) and resistance (FR) (**B**) scores in TCGA pan-solid cancer cohort. (**C**), Significant difference (by one-way ANOVA) of the FS gene signature score between *IDH1/2*-mutant and wild-type LGG (Lower Grade Glioma). (**D**), Significant difference (by one-way ANOVA) of the FR gene signature score between *KEAP1*-mutant and wild-type LUAD (Lung Adenocarcinoma).

Next, we applied the newly curated ferroptosis sensitivity (FS) and resistance (FR) gene signatures to a pan-cancer cohort (*n* = 9011) in TCGA whereby transcriptomic and clinical data are available. Low grade glioma (LGG) displays the highest FS but lowest FR score across the solid cancers (Figure 4A,B), indicating that LGG might be particularly susceptible to ferroptosis-inducing agents. Gliomas with mutations in IDH (isocitrate dehydrogenase), which leads to loss of its normal enzymatic function and the abnormal production of oncometabolite 2-hydroxyglutarate, represent a unique subset genetically and clinically distinct from that carrying wild-type IDH, particularly in LGG [27]. Importantly, we found that *IDH1/2*-mutant LGG was associated with a significantly higher FS score than *IDH1/2-*wild-type LGG (Figure 4C), prioritizing an innovative strategy to target *IDH1/2*-mutant LGG. Supporting our finding, recent studies showed that accumulation of oncometabolite 2-hydroxyglutarate, the product of the mutant IDH, sensitizes cells to ferroptosis [28] and that shRNA-based knockdown of *IDH2* increases the sensitivity to Erastin-induced ferroptosis [29].

**Figure 5:**
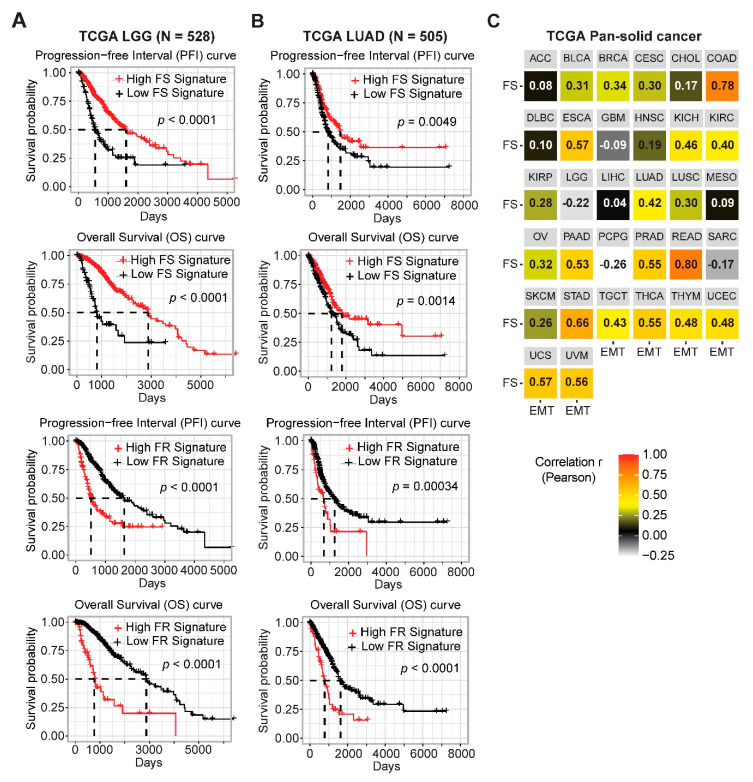
Prognostic values of the ferroptosis gene signature in cancer patients. (**A**,**B**), Kaplan–Meier survival analyses of LGG (**A**) and LUAD (**B**) stratified by the FS and FR gene signatures. (**C**), Correlation analysis of the epithelial-to-mesenchymal transition (EMT) and FS gene signatures grouped by cancer types across TCGA pan-cancer cohort.

In contrast, lung adenocarcinoma (LUAD) exhibits the highest FR score (Figure 4B), suggesting that aberrant blockage of ferroptosis might be a key feature in LUAD. The KEAP1-NRF2 axis is well known to negatively regulate ferroptosis, and cancer cells with *KEAP1* mutations are associated with increased resistance to ferroptosis [16]. In line with this notion, interrogation of a LUAD cohort in TCGA revealed that tumors with *KEAP1* mutations, frequent in LUAD samples, display significantly higher FR signature scores (Figure 4D). Importantly, a high FS score is associated with significantly better overall survival (OS) and progression-free interval (PFI), while a high FR with poor OS and PFI in patients with LGG and LUAD (Figure 5A,B), verifying the clinical relevance of the FS and FR gene signatures. 

Moreover, a previous study associated susceptibility to ferroptosis with a mesenchymal cell state [18]. Supporting this notion, we identified *ZEB1* as one of the most strongly negatively correlated genes with RSL3, Erastin, ML162 and ML210 (Figure 1B,C and Figure S1A,B). We thus further investigated the link between EMT and FS signatures across the pan-cancer cohort in TCGA, and found a positive correlation between these two phenotypes in most cancer lineages (Figure 5C). 

**Figure 6:**
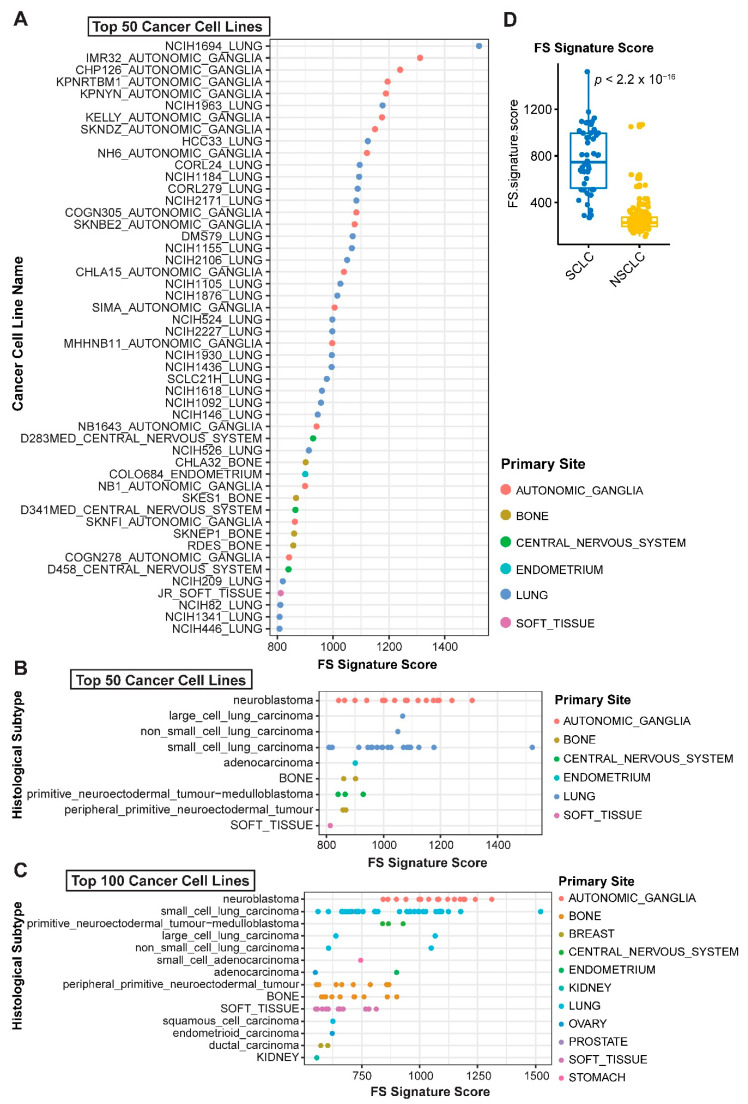
Predictive values of ferroptosis gene signature across pan-cancer cell lines. (**A**–**C**), Ferroptosis sensitivity was scored across solid pan-cancer cell lines based on the newly curated ferroptosis sensitivity gene signature. Normalized transcriptomic data were downloaded from CCLE project. (**D**), Significant difference (by unpaired two-sided t-test) of the FS gene signature score between small cell lung cancer (SCLC) and NSCLC.

To seek additional evidence supporting the applicability of the newly curated FS and FS signatures (Table S3), we prospectively probed ferroptosis response in a large cohort of pan-cancer cell lines from CCLE database (*n* = 890). Tumor cells of histological origins from small cell lung cancer (SCLC), which was not included in TCGA project, and sympathetic nervous tissue (autonomic ganglia cells) dominate both the top 50 and top 100 cell lines based on their FS scores, with the highest and second-highest FS signature, respectively (Figure 6A–C). SCLC cells have significantly higher FS scores than NSCLC (*p* < 2.2 × 10^−16^) (Figure 6D), consistent with the results derived from cancer patients that LUAD and LUSC (lung squamous cell carcinoma), two major types of NSCLC, show low FS but high FR scores (Figure 6A). Similarly, pheochromocytoma/ paraganglioma (PCPG) cancer, with the same origin as autonomic ganglia cells, exhibits the second-highest FS signature in patients of pan-cancer (Figure 4A). These results reinforce the applicability and reliability of the FS/FR gene signature and further suggest that SCLC, a highly aggressive neuroendocrine lung cancer lacking targeted therapies, might particularly benefit from ferroptosis-based therapeutics.

**Figure 7:**
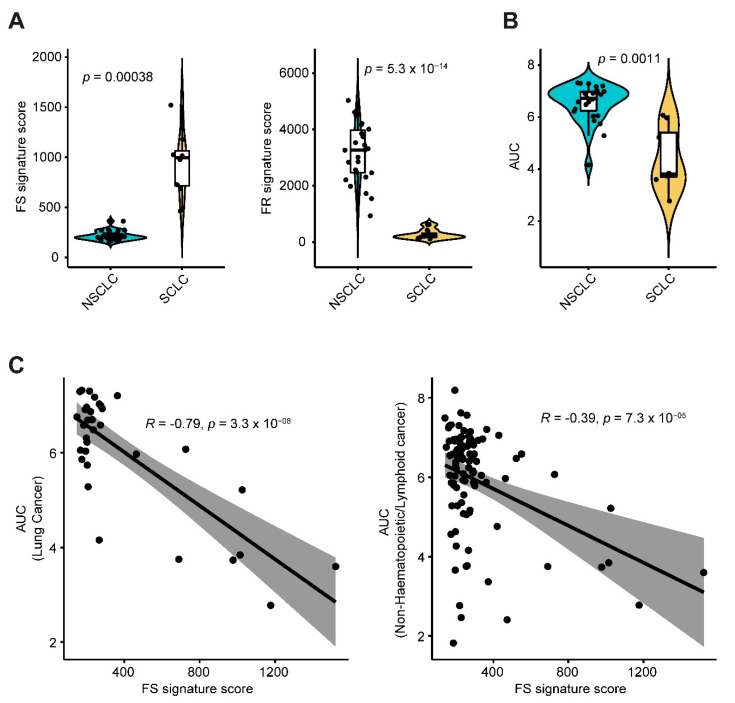
FS and FR gene signatures predict cancer cell responses to Erastin. (**A**), Violin plot shows a significant difference (by unpaired two-sided t-test) of ferroptosis sensitivity (FS) and resistance (FR) signature scores between small cell lung cancer (SCLC) and Non-SCLC (NSCLC). Normalized transcriptomic data of these cell lines were downloaded from Cancer Cell Line Encyclopedia (CCLE) project (https://portals.broadinstitute.org/ccle). FS and FR gene signatures were scored across a panel of non-hematopoietic/lymphoid-derived cancer cell lines (*n* = 99) treated with Erastin, which were downloaded from an independent study cohort (Reference 4). (**B**), Violin plot shows a significantly lower AUC (area under the curve) value in SCLC in response to Erastin than that of NSCLC (by unpaired two-sided t-test)**.** Of note, lower AUC values indicate more sensitivity or less resistance to Erastin. AUC values of Erastin were downloaded from an independent study cohort (Reference 4). (**C**), A significantly negative correlation between FS score and AUC of Erastin across lung cancer and non-hematopoietic/lymphoid-derived cancer cell lines.

Finally, we applied the ferroptosis gene signatures (Figure 3A,B) to another independent study cohort of non-hematopoietic/lymphoid cancer cell lines (*n* = 99) treated with Erastin [5]. SCLC cell lines showed significantly higher FS but lower FR signature scores than NSCLC cells (Figure 7A; Table S4). Importantly, sensitivity profiling revealed significantly lower AUC values of Erastin in SCLC than NSCLC cells (Figure 7B), indicating that SCLC cells are endowed with greater sensitivity to Erastin than NSCLC, which is in line with our results obtained from the analysis of CCLE project (Figure 6A–C). Strikingly, the FS signature score was significantly negatively correlated with the AUC value of Erastin across lung cancer (Pearson r = −0.79, *p*-value = 3.3 × 10^−08^) and non-hematopoietic/lymphoid-derived cancer cell lines (Pearson r = −0.39, *p*-value = 7.3 × 10^−05^) (Figure 6C), demonstrating that cancer cells with a higher FS signature are indeed characterized by increased susceptibility to the ferroptosis inducer Erastin.

Together, our work identified new cancer drugs with the potential to relaunch ferroptosis and delineated the gene networks associated with responses to ferroptosis in pan-cancer. The applicability and credibility of our findings are demonstrated by a multitude of lines of evidence from independent study cohorts of cancer cell lines and cancer patients. 

## 3. Discussion

Ferroptosis has increasingly gained attention due to the critical roles in tumorigenesis and cancer progression [1,8,10,18]. Despite some progress [5,6,7,9,11], the complexity of ferroptosis, especially the regulatory networks governing ferroptosis, remains enigmatic, limiting the success of ferroptosis-based therapy. Here, we provide a systematic analysis of the gene networks linked with ferroptosis, and reported on the identification of novel cancer drugs and gene clusters regulating sensitivity and resistance to ferroptosis. Our findings are supported by previous studies and clinical evidence. Informed by these findings, we show, for the first time, that the effect of ROS-related cell death induced by class I HDAC inhibitors may relate to ferroptosis, and that targeting class I HDAC with the clinically approved Vorinostat enhances the anti-proliferative effect of Erastin that is known to induce ferroptosis [30]. Besides, our results suggest that LGG, neuroendocrine SCLC, and tumors derived from sympathetic nervous tissue might particularly benefit from ferroptosis-activating agents.

Among the drug candidates with the potential to induce ferroptosis, some are not unexpected, e.g., those targeting fatty acid biosynthesis, MDM2-p53 signaling, and PI3K-mTOR pathway that have been previously reported [17,18,22]. Interestingly, we identified inhibitors of class I HDAC (Table S2), for which a link with ferroptosis has not been appreciated. Our finding, however, may explain the underlying mechanisms of ROS-related cell death conferred by class I HDAC inhibitors [23,24,25]. 

Our data also shed light on ferroptosis regulation in cancer. Of particular note, p53 [17], autophagy [16], and endoplasmic reticulum (ER) stress-associated unfolded protein response (UPR) [2,31] have been implicated in ferroptosis. Consistently, several genes identified in the present study are well-known effectors of the reported pathways: PPM1D is a downstream effector of p53, TMEM74 (transmembrane protein 74) and SQSTM1 (Sequestosome-1) involved in autophagy and DNAJC3/DNAJB14 (DnaJ homolog subfamily C/B member 3 or 14) in ER stress/UPR. The top candidacy of ELF1 (ETS-related transcription factor Elf-1) and TFAP2C (transcription factor AP-2 gamma) suggests that the two transcription factors might also play a regulatory role in ferroptosis. Collectively, we identify new compounds and gene networks regulating ferroptosis in cancer, although the underlying mechanistic insights remain to be explored.

Ferroptosis also plays a critical role in many other malignancies beyond cancer, particularly in neurodegenerative diseases [32]. It is well-known that the nuclear factor E2 related factor 2 (NRF2/NFE2L2) plays a key role in neurodegenerative disease and ferroptosis regulation. In line with this, we showed that the KEAP1-NRF2 pathway is significantly associated with a high score of FR signature (Figure 4D). It has been shown that the expression of NRF2 and its target genes declines with aging and neurodegenerative diseases [32] and emerging evidence supports a role of ferroptosis and NRF2 regulatory networks in the pathogenesis of neurodegenerative diseases [32,33,34].

Hormesis, the paradoxical beneficial effects of low-dose stressors, can be defined as the biphasic dose-effect or time-effect relationship for any substance, which is an important issue for redox-dependent aging-associated neurodegeneration/neuroprotection [33,34,35,36]. Many compounds can function through hormesis by regulating redox status and biological process in neuroprotection [34,35,37,38]. Particularly, mild stressors such as Ginkgo Biloba and polyphenol compounds, which are well-characterized stressor agents, may have beneficial effects in a hormetic-like manner by activating the NRF2-mediated stress response pathway to enhance brain neuroplasticity and increase lifespan [35,38,39,40]. Given the interconnection of NRF2 and ferroptosis, this scenario suggests a potential link of hormesis in the regulation of ferroptosis and vice versa [38,39]. 

Taken together, the identification of novel cancer drugs and gene signatures modulating ferroptosis response provides a framework to delineate the molecular mechanisms of ferroptosis regulation and to stratify cancer subsets for precision oncology. 

## 4. Materials and Methods

### 4.1. Cell Culture, Drug Treatment and Cell Viability Assay

Non-small cell lung cancer (NSCLC) cell lines (H1650, HCC827, PC9) were cultured in RPMI-1640 (Sigma-Aldrich, St. Louis, MO, USA), supplemented with 10% fetal bovine serum (FBS) (Life Technologies, Grand Island, NY, USA) and 1% penicillin/streptomycin (Sigma-Aldrich) at 37  °C in a humid incubator with 5% CO_2_. All cancer cell lines were obtained from ATCC (American Type Culture Collection, Manassas, VA, USA) and have been authenticated using STR profiling within the last three years and are confirmed free from mycoplasma contamination (Microsynth, Bern, Switzerland). Erastin (Cat. # CS-1675) and Vorinostat (Cat. # CS-0589) were purchased from ChemScene (Monmouth Junction, NJ, USA).

Cells were seeded in 6-well plates (50,000 cells/well), and treated with DMSO (control), Erastin (1 µM), Vorinostat (HDAC inhibitor, 1 µM), alone or in combination for 72 h, and cell viability was quantified by counting cell number.

### 4.2. Flow Cytometry-Based Measurement of Lipid Peroxides and Apoptosis

NSCLC cells (H1650) treated for 24 h with DMSO (control), Erastin (1 µM), and Vorinostat (HDAC inhibitor, 1 µM), alone and in combination, were subsequently stained with the Annexin V Apoptosis Detection Kit-FITC (Cat. #88-8005; Thermo Fisher Scientific, Waltham, MA, USA) according to the manufacturer’s instruction and as previously described [41,42,43]. In the meantime, some of the treated H1650 cells were washed and resuspended in 5% FBS (in HBSS), followed by staining (45 min, 37 °C) with 5 µM of BODIPY™ 581/591 C11 (Invitrogen, D3861). Flow cytometry analysis of three independent experiments was performed by BD LSRII, with data analysis by FlowJo v10. 

### 4.3. Databases

Processed drug screening and gene expression data across a set of small-molecule compounds (*n* = 481) and solid cancer cell lines (*n* = 659) from a published study [20] were downloaded for reanalysis. Correlation data across all 481 small molecules against individual transcriptomes that are significantly correlated with response to at least one small molecule were included for analysis [20]. The area under the curve (AUC), determined by fitted concentration-response curves (2-fold dilution, over a 16-point concentration range), is used as a measure of sensitivity. Fisher’s z-transformation was applied to the correlation coefficients to adjust for (normalize) variations in cancer cell line number across small molecules and contexts [20]. For validation analysis, the sensitivity profiling to Erastin of an independent cohort of cancer cell lines (*n* = 117, including 99 non-hematopoietic/lymphoid-derived cancer cell lines) [5] was employed. The publicly available database FerrDb (http://www.zhounan.org/ferrdb/) [26] was used as a reference for the newly identified biomarkers of ferroptosis. Drug-gene interaction database (http://dgidb.org/) was examined for druggable genes. Genetic landscape of identified genes across The Cancer Genome Atlas (TCGA) Pan-cancer cohort was downloaded from cBioPortal (https://www.cbioportal.org/). Normalized transcriptomic data were downloaded from Cancer Cell Line Encyclopedia (CCLE) project (https://portals.broadinstitute.org/ccle). Protein interactions and pathway enrichment analyses were based on STRING databases (version 11.0; https://string-db.org/). R software (version 3.6.3) was used for statistical analyses and data presentation.

### 4.4. Ferroptosis Gene Signatures and Patient Survival Analysis

The gene signatures of ferroptosis sensitivity and resistance were scored as the sum of the respective gene sets after scaling, respectively. Curated EMT gene signature was based on previous studies, scored as the sum of a mesenchymal gene set (*FN1  +  VIM  +  ZEB1  +  ZEB2  +  TWIST1  +  TWIST2  +  SNAI1  +  SNAI2  +  CDH2*) minus that of epithelial genes (*CLDN4  +  CLDN7  +  TJP3  +  MUC1  +  CDH1*) [44,45]. 

Survival analysis was performed using “survminer” and “survival” R packages. Transcriptomic data of primary tumor samples and clinical data of matched patients in the TCGA Pan-cancer cohort were used for survival analysis, whereby patients were divided into two groups based on a best-separation cut-off value of FS/FR gene signatures to plot the Kaplan–Meier survival curves [46,47].

### 4.5. Statistical Analysis

Data were presented as mean  ±  s.d., with the indicated sample size (*n*) representing biological replicates. Data analysis was performed by GraphPad Prism 7 (GraphPad Software, Inc., San Diego, CA, USA). Gene expression and survival data derived from the public database, as well as correlation coefficient (Pearson and Spearman), were analyzed using R (version 3.6.2) [46]. Statistical significance was determined by one-way/two-way analysis of variance (ANOVA), Bonferroni’s multiple comparison test, and Student’s t-test using GraphPad Prism 7, unless otherwise indicated. *p*  <  0.05 was considered statistically significant.

## 5. Conclusions

The identification of cancer drugs with the potential to induce ferroptosis and gene signatures predictive of ferroptosis sensitivity and resistance sheds light on ferroptosis regulatory networks and may facilitate biomarker-guided stratification for ferroptosis-based therapy. Our work thus warrants further studies to verify the drug vulnerabilities and stratification approaches. 

## Supplementary Materials

The following are available online at https://www.mdpi.com/2072-6694/12/11/3273/s1, Figure S1: A pharmacogenomic analysis identified biomarkers associated with ferroptosis inducers. Figure S2: The interaction map and pathway enrichment of ferroptosis sensitive and resistance genes. Table S1: Drug compounds whose effects (AUC values) significantly (empirical *p*-value < 0.01) positively correlated with *SCL7A11* mRNA. Table S2: Pathway enrichment analysis based on annotated targets of newly identified ferroptosis-inducing compounds. Table S3: Ferroptosis sensitivity and resistance signature scores across solid cancer cell lines. Table S4: Ferroptosis sensitivity and resistance signature scores across an independent study cohort (Reference 4).
