# Supplementary material for: Pharmacotranscriptomic Analysis Reveals Novel Drugs and Gene Networks Regulating Ferroptosis in Cancer
Source: Cancers (Basel). 2020 Nov 5;12(11):3273. doi: 10.3390/cancers12113273 (PMC7694346; doi:10.3390/cancers12113273)
Supplement: Supplementary file 1 [file cancers-12-03273-s001.zip › cancers-989570-supplementary/cancers-989570-supplementary.docx]

**Table S1.** Drug compounds whose effects (AUC values) significantly (empirical *p*-value < 0.01) positively correlated with *SCL7A11* mRNA.

**Table S2.** Pathway enrichment anaylsis based on annotated targets of newly identified ferroptosis-inducing compounds.

**Table S3.** Ferroptosis sensitivity and resistance signature scores across solid cancer cell lines.

**Table S4.** Ferroptosis sensitivity and resistance signature scores across an independent study cohort (Reference 4).


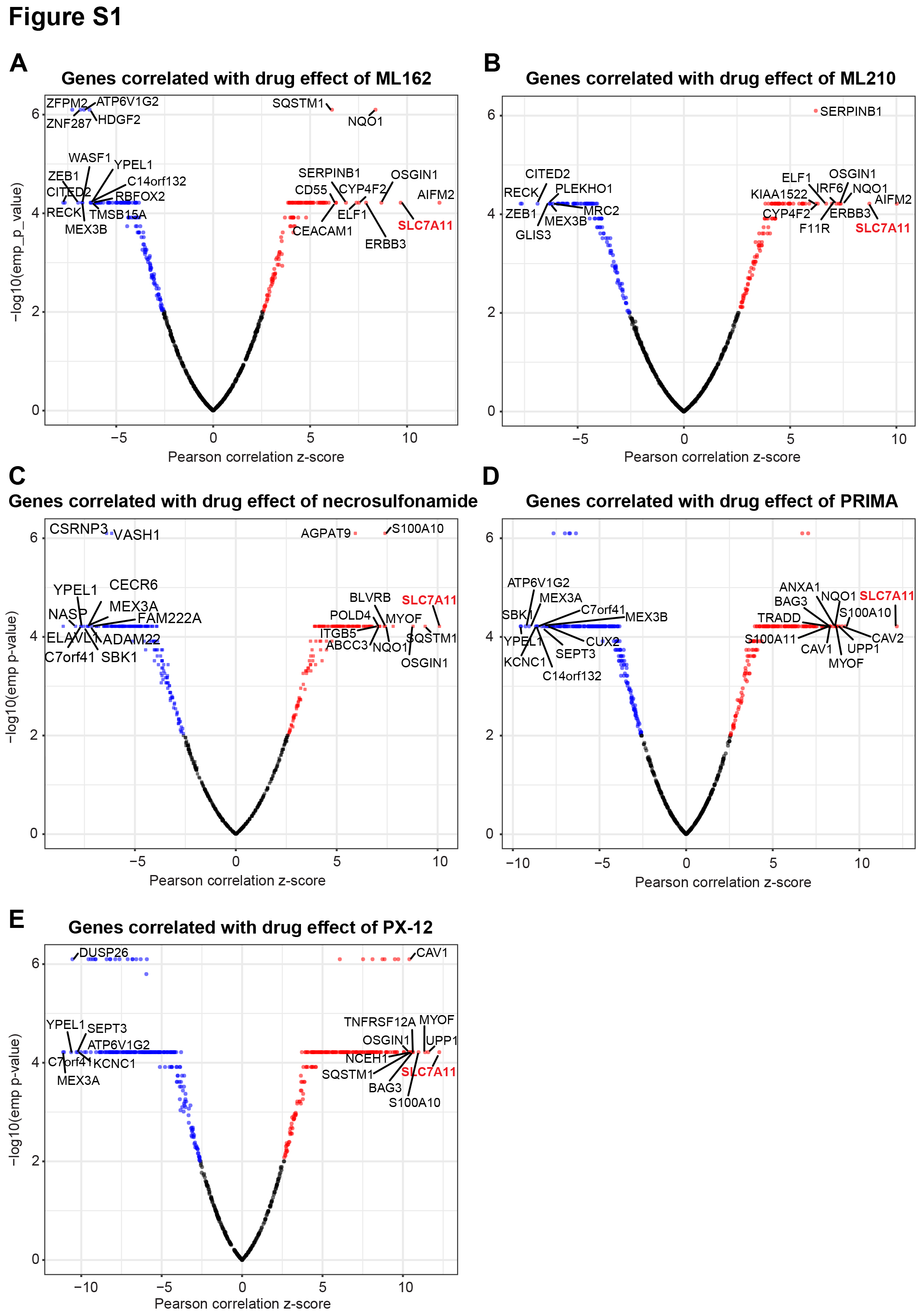


**Figure S1.** A pharmacogenomic analysis identified biomarkers associated with ferroptosis inducers. **A–E,** Correlating sensitivity profiling of the indicated ferroptosis-triggering drugs with transcriptomic gene expression across solid cancer cell lines (*n* = 659). Blue and red dots indicate negatively and positively correlated genes, respectively. Here, negative correlation indicates that a larger area under the curve (AUC), determined by fitted concentration-response curves (2-fold dilution, over a 16-point concentration range), is correlated with lower gene expression, and vice versa.


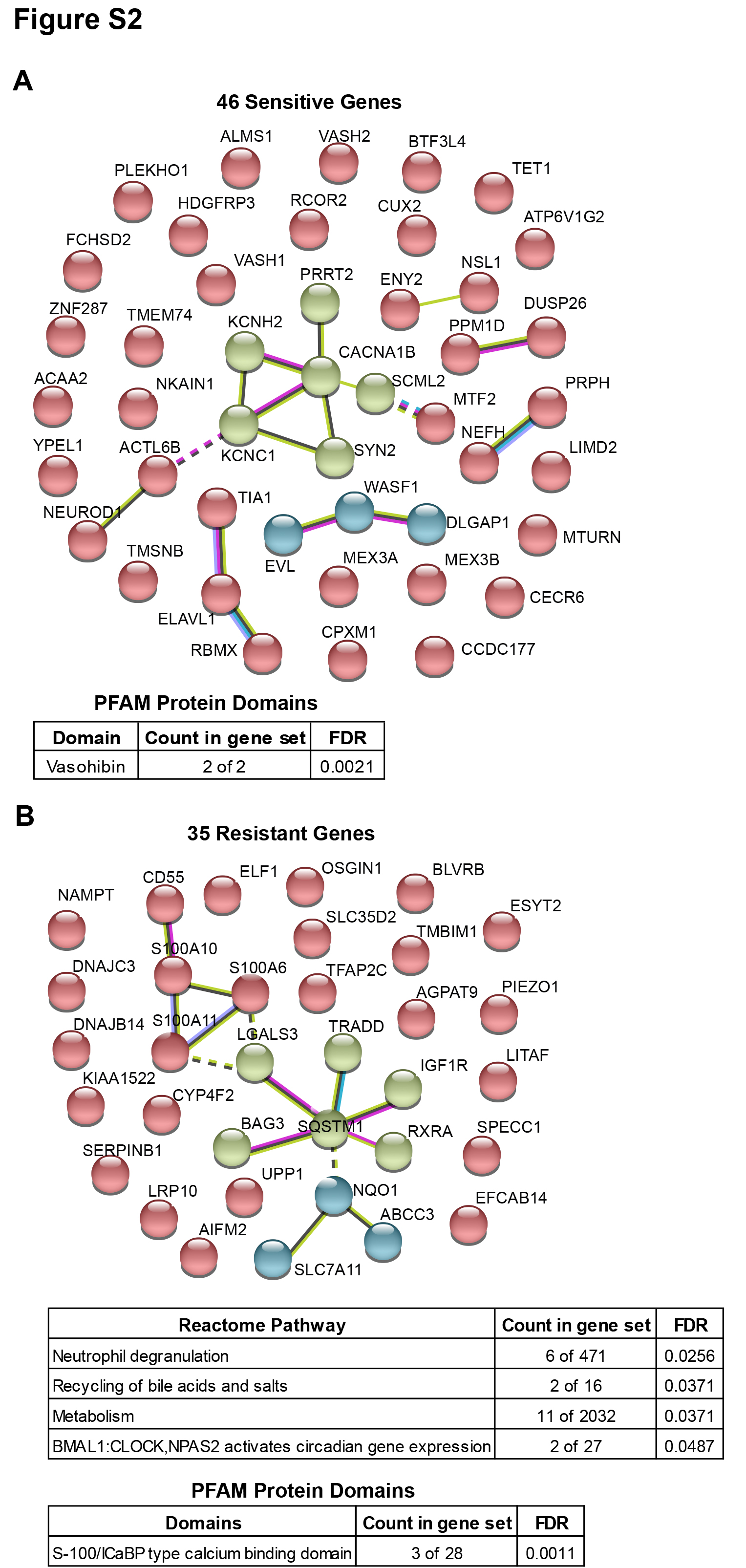


**Figure S2.** The interaction map and pathway enrichment of ferroptosis sensitive and resistance genes. **A–B,** The interaction map (upper panel), significantly (false discovery rate [FDR] <0.05) enriched pathway and protein domain (lower panel) of the genes in the ferroptosis sensitive (**A**) and resistant (**B**) group, based on STRING databases (version 11.0; <https://string-db.org/>). **C,** Kaplan-Meier survival analyses of LUAD stratified by the FS (ferroptosis sensitivity) and FR (ferroptosis resistance) gene signature scores.
